# Quality of life following third molar removal under conscious sedation

**DOI:** 10.4317/medoral.17677

**Published:** 2012-08-28

**Authors:** Manuel Sancho-Puchades, Eduard Valmaseda-Castellón, Leonardo Berini-Aytés, Cosme Gay-Escoda

**Affiliations:** 1 Fellow of the Master of Oral Surgery and Implantology. Faculty of Dentistry. University of Barcelona; 2 Professor of Oral Surgery. Professor of the Master of Oral Surgery and Implantology. Faculty of Dentistry. University of Barcelona. UB-IDIBELL Institute Researcher; 3 Professor of Oral and Maxillofacial Surgery. Subdirector of the Master of Oral Surgery and Implantology. Faculty of Dentistry. University of Barcelona. UB-IDIBELL Institute Researcher; 4 Chairman of Oral and Maxillofacial Surgery. Director of the Master of Oral Surgery and Implantology. Faculty of Dentistry. University of Barcelona. UB-IDIBELL Institute Coordinator Researcher. Head of the Oral and Maxillofacial Surgery Department of the Teknon Medical Center, Barcelona

## Abstract

Aim: The aim of this study was to assess quality of life (QoL) and degree of satisfaction among outpatients subjected to surgical extraction of all four third molars under conscious sedation. A second objective was to describe the evolution of self-reported pain measured in a visual analogue scale (VAS) in the 7 days after extraction. Study design: Fifty patients received a questionnaire assessing social isolation, working isolation, eating and speaking ability, diet modifications, sleep impairment, changes in physical appearance, discomfort at suture removal and overall satisfaction at days 4 and 7 after surgery. Pain was recorded by patients on a 100-mm pain visual analogue scale (VAS) every day after extraction until day 7. Results: Thirty-nine patients fulfilled correctly the questionnaire. Postoperative pain values suffered small fluctuations until day 5 (range: 23 to 33 mm in a 100-mm VAS), when dicreased significantly. A positive association was observed between difficult ranked surgeries and higher postoperative pain levels. The average number of days for which the patient stopped working was 4.9. Conclusion: The removal of all third molars in a single appointment causes an important deterioration of the patient’s QoL during the first postoperative week, especially due to local pain and eating discomfort.

** Key words:**Third molar removal, quality of life, sedation.

## Introduction

Although the dental profession has witnessed a dramatic reduction in dental disease during the last century, the problems associated with the third molars still persist (1). Many people require the extraction of third molars at some time in their lives, mostly due to pain, caries or periodontal problems (2-4). At this point, patients have to confront the setback of undergoing several surgical appointments, which generate a non-negligible amount of anxiety, certain postoperative morbidity and an undeniable loss of time. These reasons make some clinicians opt to perform the four extractions at a single appointment, thus merging the individual postoperative periods in one. The use of conscious sedation, as a complement to local anesthesia, may further improve the surgery by increasing both patient’s and surgeon’s comfort (5).

In the past, third molar dental literature had focused on extraction criteria and its complications (6), however more and more studies are addressing the influence third molar surgery has on patients’ quality of life (QoL) on the postoperative period (7-20). QoL is a multidimensional concept that refers to the patient’s ability to enjoy normal life activities. It is difficult to measure because it means different things to different people (12). However, questionnaires designed to measure quality, effectiveness and efficiency of treatment approaches as well as physical, social and psychological consequences of health states have been developed, providing significant information on how treatments can affect life quality (21). Understanding the impact surgery has on the patient’s life is certainly significant since patients expect the surgeon to explain risks and benefits of the planned procedure, as well as details from the recovery period.

The aim of our study was to assess QoL and degree of satisfaction among outpatients subjected to surgical extraction of all four third molars under conscious sedation. A second objective was to describe the evolution of self-reported pain measured in a visual analogue scale (VAS) in the 7 days after extraction.

## Material and Methods

Healthy patients (ASA I or II) beyond 16 years of age requiring surgical removal of all four third molars were consecutively selected. At the first appointment, the purpose of the intervention was explained, clarifying all possible complications and the anticipated postoperative course. All patients signed an informed consent. Several weeks later the four third molars were removed at a single appointment. All surgeries were performed under local anesthesia (4% articaine with 1:100.000 epinephrine) complemented by conscious endovenous sedation (2 gr midazolam, 200 mg/h propofol and 50 mg fentanyl). Intraoperatively, antibiotic, analgesic and antiinflammatory prophylaxis were administered endo-venously (2 g / 200 mg amoxicillin/clavulanic acid, 25 mg dexketoprofen and 125 mg metilprednisolone). All surgical material used was sterile. Just before surgery, patients rinsed with 0.12% clorhexidine digluconate for 1 minute. The surgical technique used was similar to that described by Leonard (22). A buccal mucoperiosteal flap was raised and protected by a Minnesota retractor. Lingual flap retraction was carried out only if necessary. Sterile low-speed hand pieces and sterile distilled water were used for ostectomy and crown sectioning. The wound was closed with 3-0 silk. Postoperative instructions included oral antibiotic and non-steroidal antiinflammatory drugs (usually 750 mg amoxicillin and 50 mg sodium diclofenac – both three times a day during 7 days), as well as 0.12% clorhexidine digluconate rinses 2 times a day for 15 days. Patients were recalled at postoperative days 3 and 7. Suture removal was performed at the seventh day appointment.

The following variables were recorded: age, gender, educational level (primary school, high school or university), professional activity (student, household, worker), and tooth position according to the Pell-Gregory classification (23). In order to calculate global surgical difficulty, each third molar received a specific score depending on its position ( Table 1). The sum of the four individual scores produced a final punctuation that ranked the surgery as simple (8-13 points), moderate (14-19 points) or difficult (20-24 points).

**Table 1:**
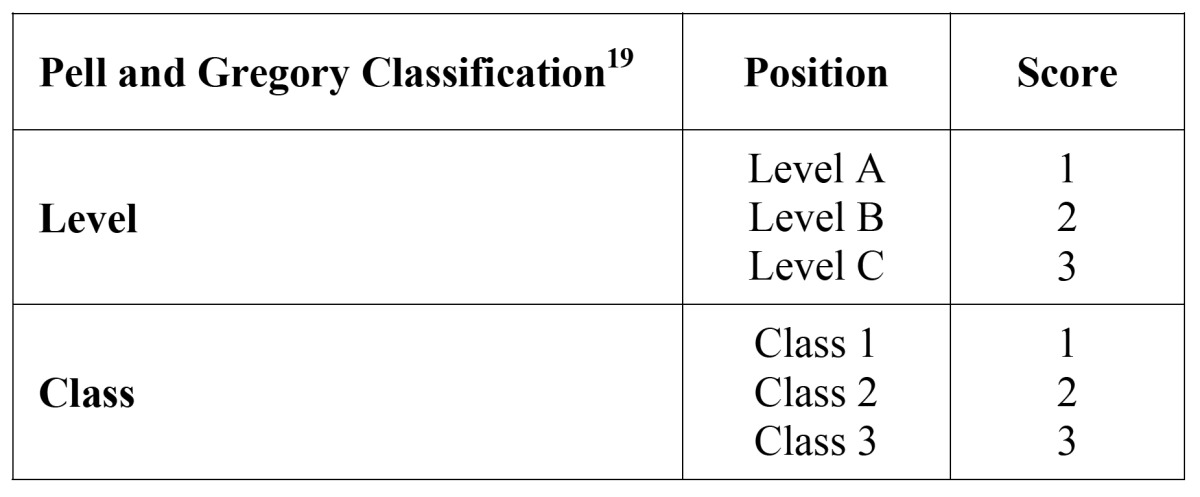
Pell & Gregory difficulty index.

Likewise, patients were given a questionnaire to fill in on day 4 after the surgery, and again on day 7, immediately after suture removal. Questionnaires were identical to those used in a previous study carried out in the same ins-titution to evaluate quality of life after the extraction of a lower third molar under local anesthesia (8). These questionnaires comprised different items addressing social isolation, working isolation, eating and speaking ability, dietetic modifications, sleep impairment and changes in physical appearance. Patients who failed to return the questionnaire were excluded from the study.

In addition, patients were asked to quantify postoperative pain every day until day 7. To this effect, a 100-mm visual analogue scale (VAS) was used.

Data were processed using the Statistical Package for Social Sciences (SPSS), version 15.0. The duration in days of the alterations was compared with t-tests for gender and professional activity and with oneway ANOVA test for level of surgical difficulty. The association of gender, professional activity and discontinuation of work was assessed with Pearson’s chisquare tests. Pain VAS scores were assessed with an analysis of variance (ANOVA) test for repeated measures with the Grenhouse-Geisser correction of the degrees of freedom if sphericity did not hold. Post hoc comparisons were made with the Bonferroni correction. In all cases, significance level was set at 0.05.

## Results

Thirty-nine out of 50 patients returned their questionnaires correctly filled and attended the follow-up visits (19 females and 20 males). Eleven patients, who either lost their questionnaire or failed to complete it correctly, were excluded from the study. The mean age was 22.3 ± 4.7 years. The mean difficulty score for the surgeries was 17 ± 2.5, which corresponds to the moderate difficulty-category.  Table 2 displays the distribution of demographical variables.

**Table 2:**
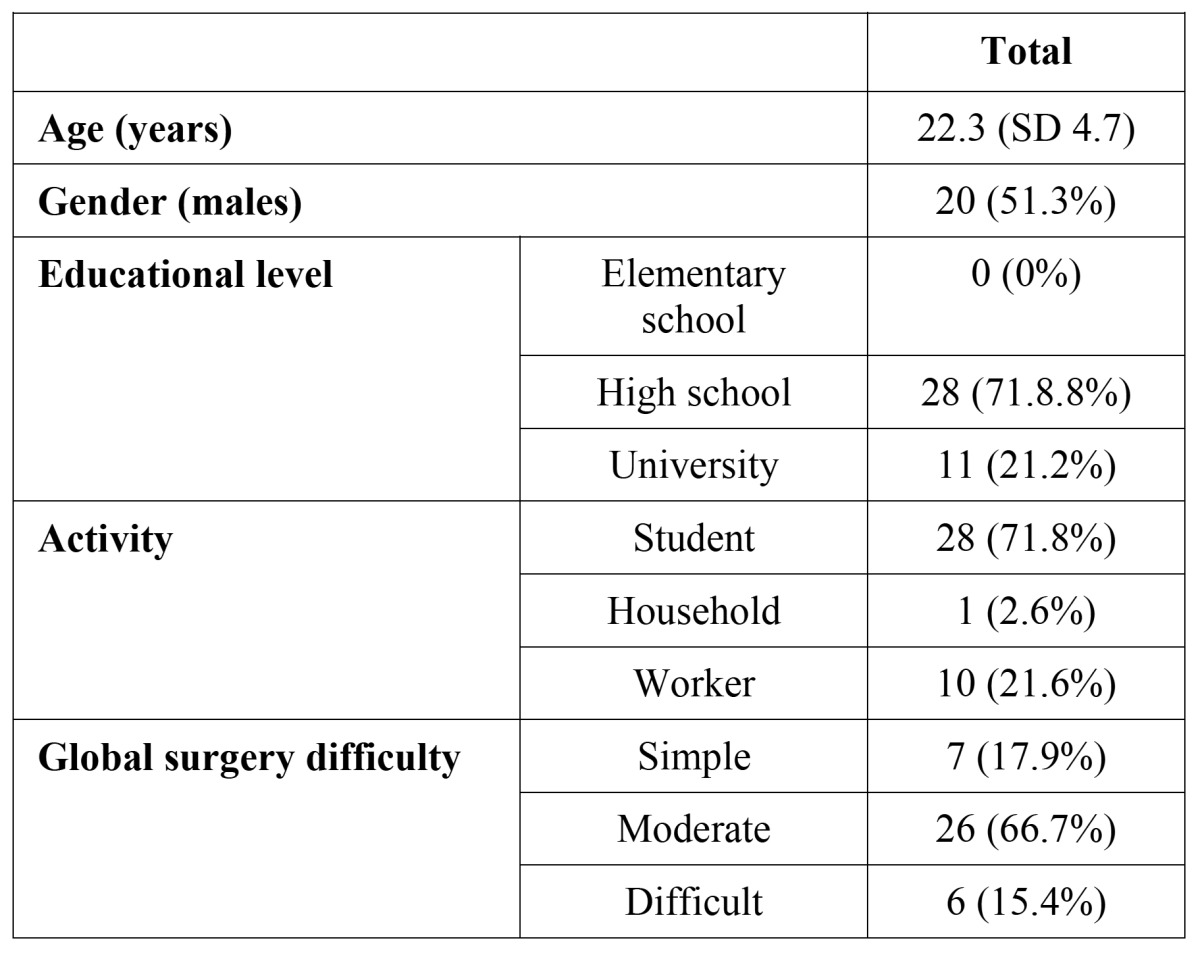
Sample distribution.

Questionnaire results are shown on  Table 3 and  Table 4.

**Table 3:**
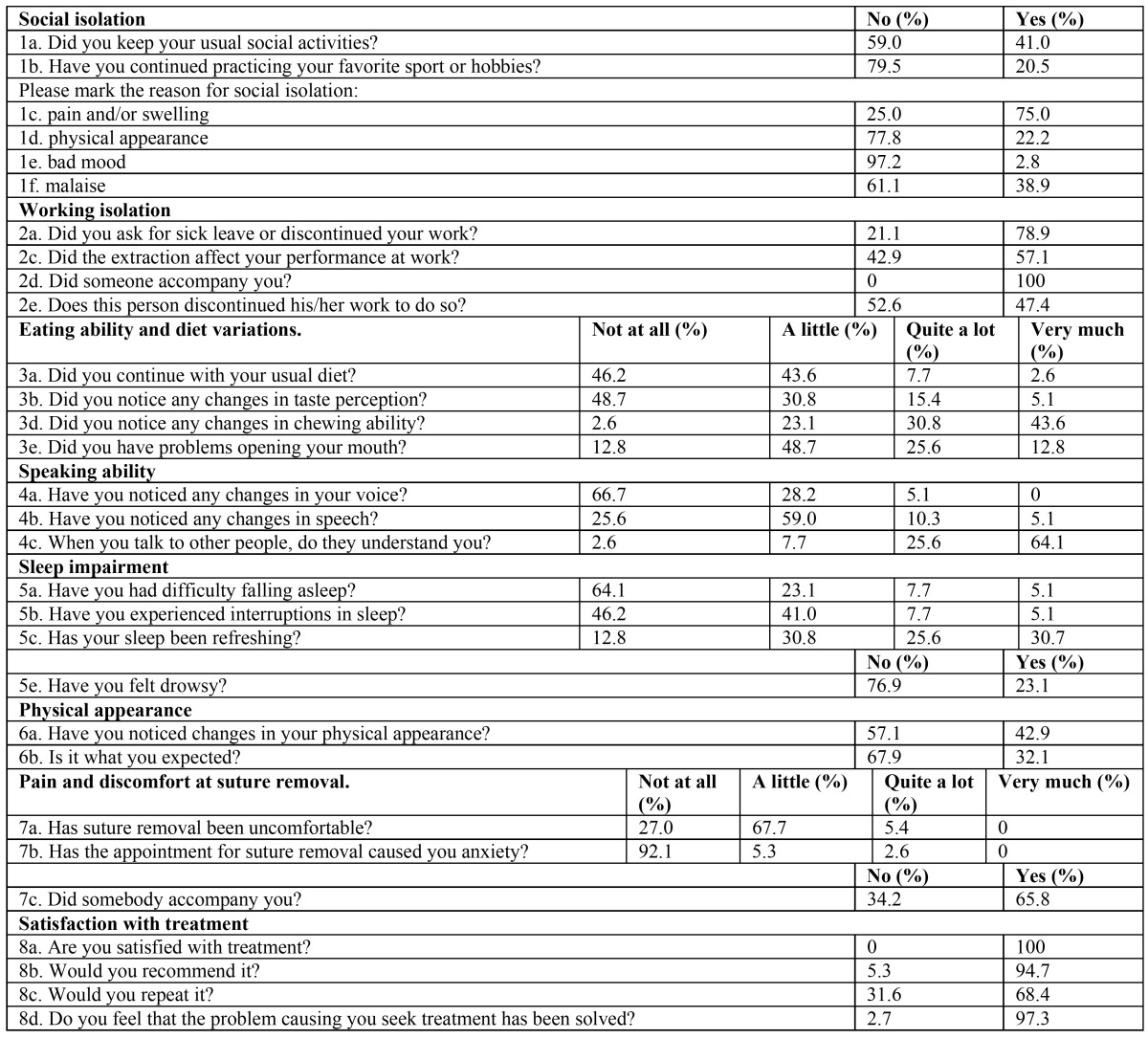
Questionnaires completed at postoperative day 4 (Questions 1-6) and day 7 after suture removal.

**Table 4:**
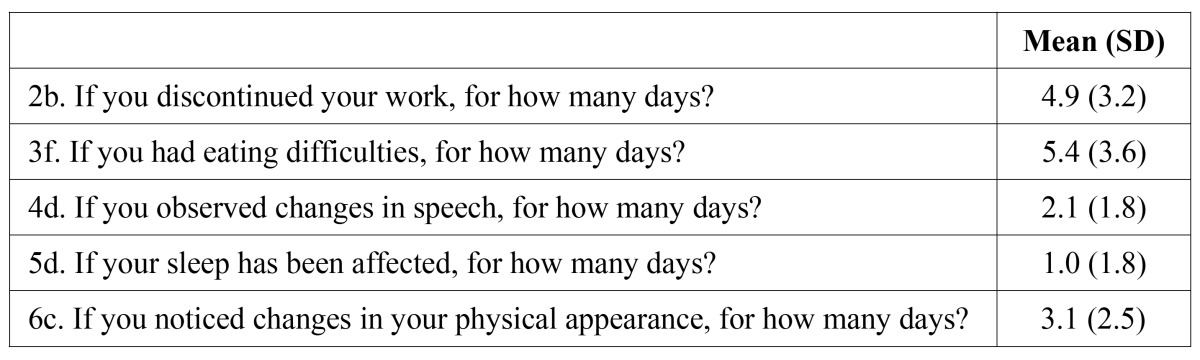
Mean duration of quality of life alterations.

**Figure 1:**
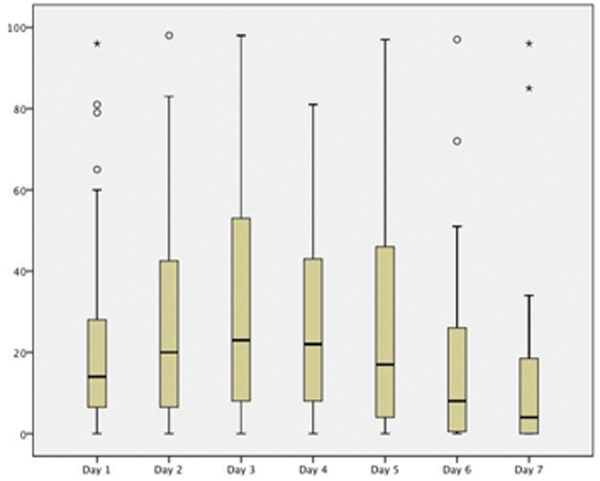
Evolution of the self-reported VAS of pain during the postoperative period. The vertical axis represents VAS scores (from 0 to 100 mm). Circles represent outlier values. Asterisks represent extreme values.

Pain VAS scores significantly varied across time (F=7.565; df=2.363; p=0,0004). VAS scores with 95 % confidence intervals are shown in figure 1. No significant differences were observed during the first five days. However on day six and seven, patients experienced a statistically significant reduction of pain (Day 6 compared with day 3 and 5: p=0.007, p=0.002 respectively / Day 7 compared with day 3, 4, 5, and 6: p=0.002, p=0.008, p=0.001, p=0.046 respectively).

There were no differences in VAS scores between male and female patients (F=0.165; df=1; p=0.687), having a similar evolution of pain values over time (F=0.087; df=2.375; p=0.942). Professional activity (student, household, worker) did not influence either the VAS scores (F=0.003; df=1; p=0.954). Conversely, the level of surgical difficulty (simple, moderate and difficult) did have an impact on pain scores (F=4.163; df=2; p=0.024). “Simple” extractions had 95 % confidence intervals of estimated marginal means between 8.02 and 37.5, “moderate” extractions ranked between 11.8 and 27.1 and “difficult” extractions between 28.7 and 60.5. Differences were only significant between moderate and difficult extractions. If the Bonferroni correction to minimize the possible type I error would not have been performed, significant differences would have aroused also between simple and difficult-ranked extractions.

No association was proved between work discontinuation and VAS scores (F=0.548; df=1; p=0.464). Pain evolution seemed similar whether the patient discontinued work or not (F=0.545; df=2.335; p=0.609). Neither, gender or professional activity, influenced postoperative sick leave (chi-square=0.033; df=1; p=0.855 / chi-square=0.711; df=1; p=0.399, respectively). In these two analyses a housewife was excluded, because she did not need a sick leave. Surgical difficulty did not influence either the days of work/study cessation (F=0.341; df=2; p=0.715).

There were no significant differences in the number of days with eating difficulty (t=-0.193; df=34; p=0.848), changes in speech (t=0.299; df=32; p=0.767), or sleep alterations (t=0.278; df=34; p=0.783) between men and women. Females noticed changes in physical appearance that were perceived to last significantly longer (t=2.101; df=34; p=0.043) than in males (4.0 days with a SD of 2.6 days versus 2.3 with a SD of 2.2 days). No differences were observed between the different surgical difficulty categories (simple, moderate, difficult) in number of days with eating difficulty (F=0.808; df=2; p=0.454), speech impairment (F=1.363; df=2; p=0.271), sleep alterations (F=0.215; df=2; p=0.808) or perceived changes in physical appearance (F=0.092; df=2; p=0.913).

## Discussion

The results of this study illustrate the considerable interference the surgical removal of all 4 third molars under local anesthesia and conscious sedation has on several aspects of patients’ daily life during the early postoperative period, especially due to local pain and interference with eating. Exploring the influence surgery has on patients’ life can be beneficial both for the patient and the surgeon. The patient will receive more truthful information in what to expect in the postoperative period and will permit him plan the best moment to undergo surgery, minimizing the impact in his life activities. On the other hand, by surveying his patients, the surgeon can evaluate the treatment approach chosen and compare the outcome with that of other techniques used (single tooth removal vs. 4 teeth removal).

However, analyzing pain perception and surgery’s impact in patients’ quality of life is always difficult due to the multifactorial nature of this process. Although the patient himself is probably the best assessor of the influence of surgery on his daily life (12,24), subjectivity is a major drawback. Variability in pain ratings of patients with the same disease or trauma is significant. Available evidence indicates that to a large extent these differences reflect individual differences in pain sensitivity (25).

Another limitation encountered in this study was that only the first 7 postoperative days were evaluated, even though some studied parameters had not reached baseline by the end of day 7. For instance, even though pain decreased significantly after postoperative day 5, the mean score at day 7 was still 12.4 in a 100-mm VAS. Similar studies comprising 4 third molar extractions in a same surgical appointment confirm that by postoperative day 7 pain scores are low but still present (9,15,16,19,20). However, both Conrad et al. (9) and White et al. (20) reported that pain scores and other studied parameters normalized around day 9, delimiting the QoL influence of these procedures to 9 postoperative days.

Interestingly enough, comparable findings have been reported when a single third molar extraction was evaluated. Colora-do-Bonnin et al.(8) found that even though there was a progressive reduction on pain intensity since postoperative day 1 (45 in the pain VAS), by day 7 mean pain scores were still 14. Our results suggest that despite a different pain fluctuation pattern, the 4 third molar extractions in a single appointment shows comparable pain values to the single tooth extraction approach, even being lower during the first two postoperative days. Due to the fact that Colorado-Bonnin’s study was elaborated in our same institution, the same surgical technique and postoperative medication was used, making measurements comparable. The only difference between both treatment protocols was that when 4 third molars were extracted, intraoperative medication was administered. The administration of intraoperative antibiotics (10), but most likely, of corticosteroids (26,27) in the 4 third molar extraction approach could explain the lower pain values recorded during the first two postoperative days in this group.

A positive association between increasing surgical difficulty and higher levels of postoperative pain has been demonstrated in this study. Nevertheless, this did not correlate with the number of days involving working isolation, eating and speaking disability or changes in physical appearance. These associations should allow the clinician give a more precise prediction of the expected postoperative period once the initial radiographic molar position diagnosis is established.

Substantial findings were observed when assessing working isolation caused by these surgeries. Nearly 80% of the patients discontinued working in the postoperative period, with a sick-leave extension of approximately 5 days. Conflictingly, other studies concerning the same surgical protocol have reported lower periods of sick leave, being around 2.5 days (7,9,18,20). Such variations could be explained as differences in patients’ pain experience or sickness perception. However, our results not support an association between postoperative pain and work cessation. Other possible justifications could be the presence of further symptoms, such as dysphagia or sleep impairment, that have shown to have a positive influence on working inability (7). Patients in our study declared having eating discomfort until postoperative day 5, what could match up with the sick-leave period experienced. On the other hand, when a single third molar is extracted the average number of days for which the patient stops working is around 1.5 days (8). Having truthful information on the expected sick leave periods of different surgical approaches will help patients choose which option fits better in their labor life.

The greatest inconvenience met by our patients was the inability to continue with a normal diet. Chewing ability and mouth opening were the main concerns. A complete incapability to continue with a normal diet by postsurgical day 4 was reported by 57.1% of our patients, while Colorado-Bonnin et al. (8) reported only 18.7%, proving that the single tooth extraction approach is compatible with a faster feeding ability recovery. Other aspects such as speaking ability and sleep impairment were not substantially altered, having little influence on postoperative discomfort. Similar results have been reported by Savin et al. (16), underlining the inability to masticate or swallow and trismus as the major nuisances during the first postoperative week.

Interpreting treatment satisfaction can be misleading. While every patient in our study admitted to be satisfied with treatment and would recommend it, 35.7% would not repeat it. Some authors interpret similar results as incorrect indications for surgery in asymptomatic patients (28). However, in our opinion, this unwillingness to repeat treatment could also be due to the distress that any surgery entails, especially during the first postoperative week. Nevertheless, the deterioration in oral health related quality of life parameters has to be considered when regarding the best third molar management option (29).

In conclusion, the removal of all third molars in a single appointment caused an important deterioration of the patient’s QoL especially during the first five postoperative days, mostly due to local pain and eating discomfort. A positive association was observed between difficult ranked surgeries and higher postoperative pain levels. The average number of days for which the patient stopped working was 4.9. The analysis of the repercussions of these surgical interventions on patients’ QoL is important for an optimal preoperative assessment and development of appropriate indications for surgery. Moreover, it enables the surgeon to give the patient realistic expectancies of the postoperative period, giving rise to a truthful informed consent and helping the patient to choose the best moment to undergo the procedure, trying to minimize major interferences with everyday life.
